# Implementation of the eHealth Appstore to improve access to self-management applications for Dutch people directly affected by cancer and their relatives: study protocol

**DOI:** 10.1007/s00520-025-09720-2

**Published:** 2025-07-05

**Authors:** A. M. de Korte, L. van Deursen, M. L. van der Lee, J. J. Aardoom, N. P. M. Ezendam, P. Heine, F. Mols, H. Mulder-Mertens, M. M. Stouten, C. R. M. Lammens

**Affiliations:** 1 The Netherlands Comprehensive Cancer Organisation, Rijnkade 5, 3511 LC Utrecht, The Netherlands; 2 CoRPS - Centre of Research on Psychological disorders and Somatic diseases, Department of Medical and Clinical Psychology, Tilburg University, Tilburg, The Netherlands; 3 Department of National Health and Care, Center for Public Health, Care and Society, National Institute for Public Health, and the Environment, Bilthoven, The Netherlands; 4 National eHealth Living Lab, Leiden, The Netherlands; 5 Scientific Research Department, Centre for Psycho-Oncology, Helen Dowling Institute, Bilthoven, The Netherlands; 6 Department of Public Health and Primary Care, Leiden University Medical Center, Leiden, The Netherlands; 7 Stichting Kanker.Nl, Amsterdam, The Netherlands; 8 Dutch Cancer Society, Amsterdam, The Netherlands; 9 National AYA ‘Young and Cancer’ Network, Amsterdam, The Netherlands

**Keywords:** EHealth, MHealth, Oncology, Self-management, Quality of life, Quality of care

## Abstract

**Objective:**

The OncoAppstore is developed to increase access and availability of reliable cancer-relevant online self-management applications for all Dutch people directly affected by cancer and their relatives, without referral. By providing health credit, the applications are available without personal costs.

**Methods:**

This study consists of three parts. Part 1 is a prospective observational study, aiming to compare characteristics of OncoAppstore users with those who participate but did not buy an application (non-users). The number and type of purchased applications and used credit will be monitored. Moreover, health-related quality of life (HRQoL) and user experience will be measured after the first application purchase or when the health credit expires (i.e. after 6 months), and 3 and 6 months thereafter. Chi-square and Mann–Whitney *U* tests compare OncoAppstore users with non-users. Part 2 is an observational study that will compare users directly affected by cancer with the general Dutch cancer population with respect to sociodemographic variables, employment, income, healthcare expenditures, and care accessibility. Differences between the two groups will be tested using Chi-square, Fisher’s exact, and Mann–Whitney *U* tests. Part 3 consists of focus groups among OncoAppstore users and non-users. The focus groups will be conducted to identify unmet needs, user preferences, usability, acceptability, and suggestions for improving the OncoAppstore. Thematic analyses will be used to analyse the qualitative data.

**Conclusion and implications:**

Data on HRQoL, healthcare expenditures, usability, and accessibility of the OncoAppstore will provide insights needed to improve the infrastructure and sustainability of self-management applications in Dutch cancer care.

## Background

Currently, over 937,000 individuals living in the Netherlands have been diagnosed with cancer in the past 20 years [1]. Trends indicate a further increase in prevalence in the forthcoming years, driven by advancements in screening and treatment leading to increased survival rates, together with the ageing population, as cancer predominantly impacts older adults [2]. It is important to recognize that the impact of cancer extends beyond those diagnosed, affecting their families and support network and thereby expanding the overall number of people affected by this disease [3].

This large and growing population often experiences a range of physical and psychological effects resulting from the disease and its treatment, such as depressive symptoms, leading to elevated psychosocial care needs [4]. These care needs place increasing pressure on the healthcare system, which is already strained by staffing shortages and rising costs [5]. However, the societal cost of cancer extends beyond healthcare, as it often compels patients and caregivers to reduce their working hours during and after treatment [6–8]. The loss of productivity can result in loss of employment or diminished work abilities. These consequences can lead to financial insecurity, reduced quality of life (QoL), and increased reliance on social security systems [6–9]. Employers also bear economic burdens, facing costs associated with absenteeism, reduced productivity, and temporary staffing [8]. This underlines the necessity of reevaluating current cancer care to ensure optimal use of limited resources. eHealth offers a promising solution by enabling greater scalability of care while maintaining quality. It can provide (cost-)effective additions and alternatives to ‘care as usual’. Additionally, eHealth interventions could play a crucial role in decreasing care demands by supporting tertiary prevention, helping to mitigate the broad societal costs and impact of cancer.

Several eHealth interventions focussing on prevention have already been developed specifically for individuals directly affected by cancer and their relatives. These interventions have shown promising outcomes in alleviating factors, such as cancer-related fatigue, pain, and depressive symptoms, and enhancing social functioning and knowledge [10–16]. However, access to and availability of some of these interventions is restricted. This is due to user costs, limited discoverability, and the requirement for a subscription and/or referral by a healthcare professional.

Between 2019 and 2021, the Dutch Ministry of Health, Welfare and Sport initiated and funded the national FitKnip experiment, providing individuals from the general population with a €100 health credit to purchase preselected, evidence-based online self-management tools. The health application platform was designed to encourage the use of trustworthy health applications, empower individuals to improve their health, and ultimately foster a healthier society. Feedback indicated strong enthusiasm from both users and stakeholders, with high satisfaction regarding the platform’s design, ease of use, and payment system. However, only 42% of participants used the budget to purchase at least one application during the 8-month experiment period. Users expressed a desire for a broader and more diverse selection of applications, along with greater personalisation in terms of language and categorisation. In its current form, the platform did not appear to significantly enhance users’ health empowerment or health outcomes [17–19]. Other stakeholders were encouraged to build on this work, guided by insights gained from the experiment.

Subsequently, the Dutch Cancer Society (KWF), Stichting Kanker.nl (cancer.nl foundation), and the Netherlands Comprehensive Cancer Organisation (IKNL) have taken the initiative to refine and implement the FitKnip experiment concept in an ‘OncoAppstore’, specifically targeting people directly affected by cancer and their relatives. The aim is to create an OncoAppstore within the Dutch online platform ‘Kanker.nl’, offering reliable cancer-specific and cancer-relevant online self-management applications. By enhancing the discoverability, accessibility, and funding, we expect to increase the usage of online self-management applications and tools—potentially reducing regular care demands and costs while increasing health-related quality of life (HRQoL). Lessons learned from the FitKnip experiment will aid implementation and uptake [17].

The current implementation study aims to assess HRQoL, healthcare expenditures, and other characteristics of OncoAppstore users compared to (i) OncoAppstore visitors who participated in the study but never purchased an application (non-users) and (ii) the general Dutch cancer population. Moreover, the study aims to identify unmet needs, user preferences, usability, acceptability, and suggestions for improving the OncoAppstore. These insights are relevant to support the improvement of the self-management application infrastructure and sustainability in Dutch cancer care.

## Methods

The study was submitted and exempt from review by the Medical Research Ethics Committee Brabant (NW2022-42), as this study is not subject to the Dutch Medical Research Involving Human Subject Act. The study adheres to the Declaration of Helsinki [20]. The study protocol is written in accordance to the Strengthening The Reporting of Observational studies in Epidemiology (STROBE) statement for cohort studies and follows the consolidated criteria for reporting qualitative research (COREQ) [21–23].

### Design

**Fig. 1:**
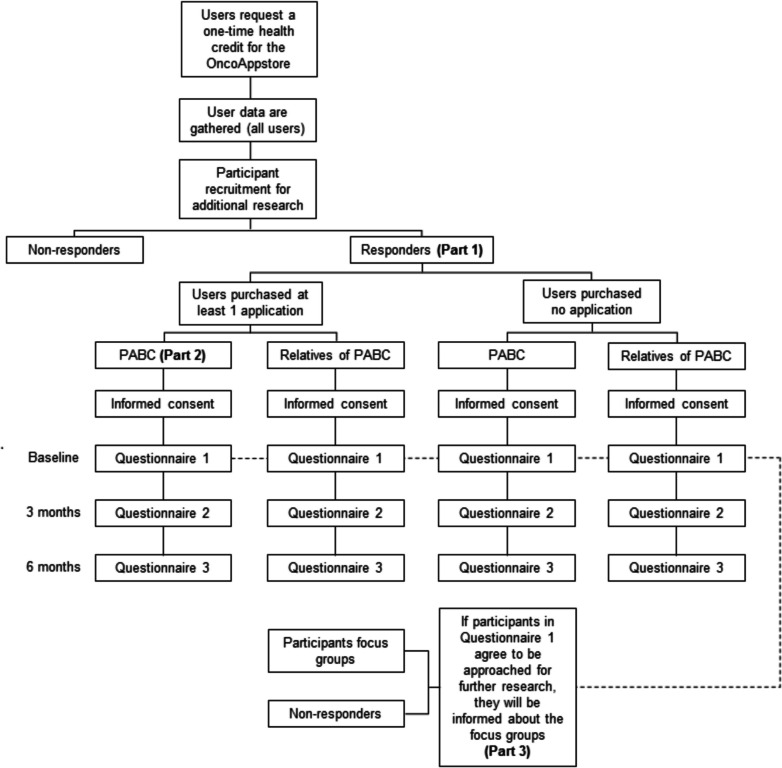
OncoAppstore study design. Abbreviation: PABC, people directly affected by cancer

This protocol paper highlights the three parts of the study (Fig. 1). Part 1 is a prospective observational study, aiming to compare characteristics of OncoAppstore users, both people directly affected by cancer and their relatives, with those who requested a health credit but did not buy or use any application while the health credit was available (non-users, both people directly affected by cancer and their relatives). Part 2 is an observational study that compares people directly affected by cancer that purchased applications from the OncoAppstore (users directly affected by cancer) with the general Dutch cancer population with respect to sociodemographic variables, employment, income, healthcare expenditures, and care accessibility. Part 3 consists of focus groups among OncoAppstore users and non-users. The focus groups will be conducted to identify unmet needs, user preferences, usability, acceptability, and suggestions for improving the OncoAppstore. A study design overview is shown in Fig. 1.

### Setting

The OncoAppstore has been developed to offer cancer-specific and cancer-relevant online self-management applications for people directly affected by cancer and their relatives. The OncoAppstore was launched within the Dutch online platform ‘Kanker.nl’, as the platform is widely known in the Netherlands among people directly affected by cancer, their relatives, and healthcare professionals. The platform has over 1,000,000 visits per month and more than 39,500 individuals with an account. The OncoAppstore is further promoted through information on websites of hospitals, cancer patient organisations, and application suppliers, (social) media campaigns, and through presentations for healthcare professionals. The OncoAppstore applications have to meet the Dutch Regional Public Health Service (in Dutch: GGD) assessment criteria pertaining to usability, reliability, privacy, and scientific foundation [24]. Consequently, project team members (AK and MS) will test each application prior to adding it to the OncoAppstore. Participants are encouraged to use the applications by providing them with an online health credit of 100 euros that is valid for 6 months, eliminating any personal costs. Additionally, a referral from a healthcare professional is not required. To purchase an application, participants will first have to create an account on ‘kanker.nl’, request the one-time health credit, and then select applications that meet their needs. There is a helpdesk available for the OncoAppstore and for specific help with an application, users can contact the supplier. Contact details are readily available in the OncoAppstore. For questions regarding the study, participants can contact the project team. Contact details are included in the participant information letter and they can contact the project team directly within the online questionnaire registry as well.

### Part 1

#### Population and recruitment

The Part 1 study population consists of Dutch-speaking adult OncoAppstore participants who requested the health credit. Not all participants will purchase an application during the 6-month period in which the health credit is valid, therefore, two groups will be formed. The first group will consist of OncoAppstore users and the second group consists of non-users. While requesting the health credit, participants will be asked if they may be approached for additional research for this study. If participants agree, they will be sent a participant information letter via the PROFILES registry [25] via email, and they will be asked to provide informed consent digitally. PROFILES is a system designed for data collection among cancer patients, encompassing everything from inviting participants to collecting data through web-based questionnaires and integrating these data with clinical data (in case of the users and non-users directly affected by cancer). By signing the consent form, people directly affected by cancer agree that their data will be linked to Statistic Netherlands [26], the Netherlands Cancer Registry (NCR), and Dutch Hospital Data [27]. After obtaining the informed consent, participants will be invited by email to fill in a web-based questionnaire (baseline). Depending on the group, this will be directly after purchasing the first application or after their health credit has expired. Thereafter, participants will be asked to complete two more questionnaires, 3 and 6 months after baseline. In the event of a non-response, a reminder email will be sent a week after the invitation email, for each questionnaire.

#### Outcome measures

*User data* will be collected from all OncoAppstore users and non-users who have received a health credit. To ensure anonymity, no personal information will be collected unless the participant explicitly agrees to be approached for addition research. The user data will include the total number of participants, as well as which applications are purchased and how frequently each application is purchased by the participants. Additionally, user-specific data will be gathered pertaining to which participant requested a health credit in the last week, whether that participant agreed to be approached for this study, the participant’s email address if he/she agreed to be approached, whether the participant purchased at least one application, which applications were purchased per user, and the amount of remaining health credit after 6 months.

Additional information will be gathered within the PROFILES registry. Data collected in the PROFILES registry will include self-reported sociodemographic (i.e. gender, age, educational level, marital status) and clinical characteristics (i.e. tumour recurrence (yes/no), co-morbidities, cancer diagnosis (yes/no), if ‘no’; type of cancer of relative, and time since diagnosis of relative). Moreover, part 1 questionnaires consist of the distress thermometer (DT) and the problem list [28], questions on employment status, work-related problems, work ability [29], user experience with the OncoAppstore, and the European Organisation for Research and Treatment of Cancer Quality of Life Questionnaire Core (EORTC QLQ-C30) [30]. Subsequently, the computerized adaptive test (CAT) version of the EORTC Core will be used instead of the 30-item version after initial data is gathered to guide the design of the EORTC CAT Core [30–32].

The *DT* is a single-item measure of distress experienced in the last week, ranging from 0 (no distress) to 10 (extreme distress) [28]. The problem list measures dichotomous responses (yes/no) on 47 items and focuses on practical problems (seven items), family/social problems (three items), emotional problems (nine items), religious/spiritual problems (two items), and physical problems (25 items). Additionally, several questions are included to assess participants’ interactions with healthcare professionals and the extent to which these interactions are related to the cancer diagnosis. Specifically, participants are asked whether they would like to speak with a specialist about their problems, whether they have already done so, and whether they have previously received help for similar issues. For the latter question, response options include ‘yes’, ‘maybe’, or ‘no’. If participants respond with ‘yes’ or ‘maybe’, they are asked to indicate from whom they received support, with multiple responses possible. Options include nurse, spiritual caregiver, dietician, psychologist, fellow patients, physical therapist, social worker, physician or oncologist, general practitioner, assistant practitioner, information and support centre, or other. Finally, participants are asked whether these visits were related to the cancer diagnosis (of themselves or a relative) or its aftermath, with the response options: ‘yes, always’, ‘yes, sometimes’, or ‘no’.

Information on employment status and work-related problems will be collected using questions about current occupation and work hours, occupation and work hours prior to cancer diagnosis, current sick leave (yes/no), and if so, is change in occupation, work hours, or sick leave caused by current disease complaints (yes/no)? Work ability will be measured by the first item from the Work Ability Index [29]. Participants will be asked to estimate their current work ability with their lifetime best on a scale from 0 (cannot work) to 10 (best ever).

In addition, participants will be asked to answer questions about user experience*.* For example, how did you find the OncoAppstore; why did you request a health credit; what do you think of the selection of applications being offered; do you find the applications you use helpful; would you recommend the OncoAppstore to others; have you experienced any technical problems; and what could be done to further encourage the use of applications?

The *EORTC QLQ-C30* Dutch version measures HRQoL and consists of a global HRQoL domain, five functioning domains (i.e. physical functioning, role functioning, emotional functioning, cognitive functioning, and social functioning), nine symptom domains (i.e. fatigue, pain, nausea and vomiting, appetite loss, dyspnoea, sleep disturbances, diarrhoea, constipation, and financial impact of disease). Each domain comprises between one and five items, with a total of 30 items [30]. In the EORTC CAT Core version, each domain comprises between seven and 34 items, with a total of 262 items in the fourteen-item bank. The questionnaires use response options on a four-point scale of ‘Not at all’, ‘A little’, ‘Quite a bit’, and ‘Very much’. The item selection from the item bank is tailored to each individual based on responses to prior items [31, 32]. Higher scores on functional health domains represent higher levels of functioning, while higher scores on the symptom scales indicate more symptom burden.

The *NCR* of the Netherlands Comprehensive Cancer Organisation will be used to link clinical data (e.g. tumour type, date of cancer diagnosis, histological classification, clinical stage, primary treatment, date of death) to part 1 participant data of people directly affected by cancer. This will be done by researchers from IKNL on the basis of personal identifiers such as date of birth, name, sex, and address. The NCR routinely collects data on all newly diagnosed cancer patients in the Netherlands. This will enable us to compare OncoAppstore participants directly affected by cancer with non-users directly affected by cancer.

#### Data analyses

Statistical analyses of the quantitative data will be performed using Statistical Analyses Software version 9.4 (SAS Institute, Cary, NC, USA), SPSS version 25 [33]. A descriptive analysis will be used to describe the sociodemographic data and clinical characteristics. These variables will be compared at baseline between the different groups using chi-square analyses or Fisher’s exact tests for categorical variables and ANOVA or Mann–Whitney *U* tests for continuous variables. Linear mixed models will be used to assess the course of HRQoL and distress over time between the two groups.

### Part 2

#### Population and recruitment

OncoAppstore users from part 1 will be included if they have been diagnosed with cancer and provided informed consent for the additional research (users directly affected by cancer). These participants will be compared to the general Dutch cancer population. People directly affected by cancer who are deceased prior to the OncoAppstore launch will be excluded from the general Dutch cancer population.

#### Outcome measures

Data from *Statistic Netherlands* [26] will be linked to the participant data collected in part 1 of this study by researchers from Statistic Netherlands. Records will be linked based on personal identifiers such as date of birth, sex, and address. Statistics Netherlands is the national statistical office of the Netherlands, responsible for collecting and publishing data on the Dutch economy, population, and society. The data are based on large-scale surveys, administrative records (e.g. municipal and tax data), and other official registers. The Statistic Netherlands data come from the System of Social Statistical Datasets, which is a system containing information on persons, jobs, benefits, and hospitalizations among others [34]. Measures from the Statistic Netherlands database that will be used are (i) income, (ii) employment status, (iii) medical care expenses, and (iv) access to health care.

Moreover, data from *Dutch Hospital Data* [27] on hospital admissions and cancer diagnoses in the Netherlands from 2018 to 2022 will be linked to the Statistic Netherlands dataset.

#### Data analyses

Statistical analyses of the quantitative data will be performed using SPSS version 25 [33] and R statistical software [35]. A descriptive analysis will be used to describe the sociodemographic data and clinical characteristics. These variables will be compared between the OncoAppstore users directly affected by cancer and the general Dutch cancer population using chi-square analyses or Fisher’s exact tests for categorical variables and ANOVA or Mann–Whitney *U* tests for continuous variables. Moreover, binary logistic regression analyses or multinominal logistic regression analyses will be conducted to investigate whether there are significant differences between the different groups based on Statistic Netherlands measures.

### Part 3

#### Population and recruitment

All research participants from part 1 will be asked to consider participating in the qualitative research of part 3. A member of the project team (AK) will inform interested participants about the focus groups by email and will send the informed consent form for part 3. After obtaining informed consent digitally, focus groups will be scheduled. The study aims to include a minimum of 20 people who are a representative group of users and non-users as well as people directly affected by cancer and relatives of people directly affected by cancer.

#### Outcome measures

A topic list will be developed by the project team for the focus groups based on the TICD checklist [36] focusing on identifying barriers and facilitators for implementation and will include the following five topics (a) unmet needs, (b) user preferences, (c) usability, (d) acceptability, and (e) suggestions for improving the OncoAppstore. Thereafter, patient experts will test the topic list and provided feedback on content and clarity of the topics presented. Semi-structured focus groups with open-ended questions will be used to encourage the participants to speak openly.

#### Data analysis

Focus groups will be audio recorded and transcribed verbatim using artificial intelligence tools and validated by two independent master students. All personal details that might appear in the transcripts will be deleted to ensure participants’ anonymity. Audio recordings will be stored until the transcripts are verified. Other data will be stored for 15 years in accordance with the legal, ethical, and quality requirements for scientific research. A thematic analysis will be applied by the project team members [37]. Barriers and facilitators for implementation will be identified from the transcripts. Approximately 10% of the transcripts will be coded independently by two project team members to develop an overall impression of the content and to develop a coding tree, using Atlas.ti version 9.0.15 (Berlin, Germany). The remaining transcripts will be coded by one project team member. Codes will then be divided into main and subthemes according to the domains of the TICD checklist and discussed by the project team until discrepancies are resolved through discussion and comparison.

## Discussion

Creating the OncoAppstore with reliable cancer-related self-management applications, which can be purchased with a provided health credit, could help improve discoverability, accessibility, and reimbursement of online self-management applications for people directly affected by cancer and their relatives. By improving access to self-management applications, reduced psychosocial consequences of the cancer diagnosis and/or treatment are expected in users of the OncoAppstore. This could reduce care demands and (societal) costs of this disease.

Participant data, clinical characteristics, and HRQoL will be gathered. Furthermore, by examining the representativeness and characteristics of the user population to the general target population, this study will provide insights into sub groups of potential users not yet reached. This could help alter and enhance future promotion and implementation of the OncoAppstore to reach a broader group of users. This is important to limit existing health disparities and provide equal access to the OncoAppstore and the health credit for all people directly affected by cancer and their relatives. Moreover, since both application users and non-application users are invited to participate in the focus groups, it will be possible to gain insight into usability, accessibility, needs, and desires of a wide variety of potential users. By collecting this data on HRQoL, healthcare expenditures, usability, and accessibility this study will not only provide insights into the user population, but it could help demonstrate the value of the online self-management applications for both users and the healthcare system. This could provide valuable evidence to support inclusion of online self-management applications for people directly affected by cancer and their relatives in reimbursement systems.

### Study limitations

A strength of the study is that the OncoAppstore is directly implemented into a real-world setting, allowing for access to self-management applications for all interested individuals without delay. Additionally, most applications within the OncoAppstore are developed in a research setting and have shown promising results in alleviating factors such as cancer-related fatigue, pain, depressive symptoms, and social functioning [10–15]. Moreover, applications will have to meet certain criteria prior to being added to the OncoAppstore thus guaranteeing a reliable selection of applications [24]. Furthermore, the OncoAppstore is run on kanker.nl, the online platform, as the platform is widely known among people directly affected by cancer, their relatives, and healthcare professionals, with over 1,000,000 visits per month and more than 39,500 individuals with an account. The study of van Deursen et al. [38] mentions unawareness of the existence of digital interventions as an important barrier to uptake of self-management applications. In addition, it was reported that patients are hesitant to pay for the interventions [38]. By eliminating costs and using a well-known platform, the OncoAppstore addresses both of these barriers. This could promote broader inclusivity among diverse groups of people directly affected by cancer and their relatives. Access to internet is unlikely to be a significant barrier, as Statistic Netherlands reported that in 2022, 98% of Dutch households had internet access, with nine out of ten individuals using it on a daily basis [39]. Another strength of this study is the implementation of EORTC CAT Core questionnaire which shows promising results in the literature. Evaluations indicate higher measurement precision and increased statistical power of the EORTC CAT Core with its 262 items compared to the 30-item version. Additionally, floor and ceiling effects were seen less often, thus creating more certainty about the ability to detect changes [40].

The study is part of an implementation project, resulting in a defined study period of 2 years not based on participant inclusion. Therefore, no sample size calculations are performed. Furthermore, participants of the OncoAppstore are not obliged to participate in the study; therefore, sociodemographic data, clinical characteristics, and the data from the questionnaires will solely be collected from a sub group of OncoAppstore users. This could introduce bias when reporting on the characteristics of the user population and when comparing users to non-users. Moreover, the study has a relatively short follow-up period of 6 months, which will hamper the possibility to detect the effectiveness of the OncoAppstore on HRQoL in the long term. Additionally, a wide variety of applications will be included in the OncoAppstore and specific instructions on the use of the applications are not provided for this study. Consequently, making it more difficult to draw conclusions on the effectiveness of specific applications and the OncoAppstore as a whole, since each participant may use the OncoAppstore and its applications differently. However, this allows participants to tailor the use and selection of applications to their personal preferences and resembles the way it will be used in the real world. Furthermore, this study targets a heterogeneous study population of people directly affected by cancer with different cancer diagnoses, stages, and treatments, as well as relatives of people directly affected by cancer. This limits the ability to draw conclusions on the OncoAppstore effectiveness in specific populations.

### Clinical implications and conclusions

Positive outcomes of this study could provide support for reimbursement of self-management applications for people directly affected by cancer and their relatives. Consequently, this study could aid optimalisation and broad implementation of accessible online self-management applications in Dutch cancer care.

## Data Availability

No datasets were generated or analysed during the current study.
